# Pan-Canadian assessment of pandemic immunization data collection: study methodology

**DOI:** 10.1186/1471-2288-10-51

**Published:** 2010-06-08

**Authors:** Jennifer A Pereira, Susan Quach, Christine Heidebrecht, Julie Foisy, Sherman Quan, Michael Finkelstein, Christopher A Sikora, Julie A Bettinger, David L Buckeridge, Anne McCarthy, Shelley Deeks, Jeffrey C Kwong

**Affiliations:** 1 Ontario Agency for Health Protection and Promotion, Toronto, Canada; 2 University Health Network, Toronto, Canada; 3 Centre for Innovation in Complex Care, Toronto, Canada; 4 Toronto Public Health, Toronto, Canada; 5 School of Public Health, University of Alberta, Edmonton, Canada; 6 Vaccine Evaluation Centre, University of British Columbia, Vancouver, Canada; 7 McGill University, Montreal, Canada; 8 Division of Infectious Diseases, Ottawa Hospital, Ottawa, Canada; 9 Department of Family and Community Medicine and Dalla Lana School of Public Health, University of Toronto, Toronto, Canada; 10 Institute for Clinical Evaluative Sciences, Toronto, Canada

## Abstract

**Background:**

The collection of individual-level pandemic (H1N1) 2009 influenza immunization data was considered important to facilitate optimal vaccine delivery and accurate assessment of vaccine coverage. These data are also critical for research aimed at evaluating the new vaccine's safety and effectiveness. Systems used to collect immunization data include manual approaches in which data are collected and retained on paper, electronic systems in which data are captured on computer at the point of vaccination and hybrid systems which are comprised of both computerized and manual data collection components. This study's objective was to compare the efficiencies and perceptions of data collection methods employed during Canada's pandemic (H1N1) 2009 influenza vaccination campaign.

**Methods/Design:**

A pan-Canadian observational study was conducted in a convenience sample of public health clinics and healthcare institutions during the H1N1 vaccination campaign in the fall of 2009. The study design consisted of three stages: Stage 1 involved passive observation of the site's layout, processes and client flow; Stage 2 entailed timing site staff on 20 clients through five core immunization tasks: i) client registration, ii) medical history collection, iii) medical history review, iv) vaccine administration record keeping and v) preparation of proof of vaccine administration for the client; in Stage 3, site staff completed a questionnaire regarding perceived usability of the site's data collection approach. Before the national study began, a pilot study was conducted in three seasonal influenza vaccination sites in Ontario, to both test that the proposed methodology was logistically feasible and to determine inter-rater reliability in the measurements of the research staff. Comparative analyses will be conducted across the range of data collection methods with respect to time required to collect immunization data, number and type of individual-level data elements collected, and clinic staff perceptions of the usability of the method employed at their site, using analysis of variance (ANOVA).

**Discussion:**

Various data collection methods were employed at immunization sites across Canada during the pandemic (H1N1) 2009 influenza vaccination campaign. Our comparison of methods can facilitate planning an efficient, coordinated approach for collecting immunization data in future influenza seasons.

## Background

Canada's preparation for an influenza pandemic included the establishment of provincial/territorial infrastructure to collect client data at the time of vaccination as well as decisions on the data elements required for national reporting of vaccination utilization. The efficient collection of client level data is vital to support optimal vaccine delivery processes, timely assessment of vaccine coverage and surveillance statistics at all population levels and the completion of research to address important questions including those regarding the new vaccine's safety and effectiveness[1].

There are three distinct systems used to collect immunization data: manual, electronic and hybrid. Manual approaches utilize paper forms to collect client immunization information at the point of care. While this is the most simple and low-cost system, it precludes the immediate availability of data. In the event that reporting on vaccine coverage is required on a weekly basis, an abstracter would need to manually audit the forms to extract the necessary data. This extra step in the reporting process leads to less timely access and could introduce considerable human error in the reported numbers. The electronic approach consists of providers directly entering client data into an electronic information system at the point of care. The costs of such a system and the staff training requirements are the two principle barriers to this approach but the benefits of rapid and accurate reporting of immunization events for decision-making, evaluation and surveillance purposes are substantial. Hybrid systems involve both paper and electronic methods, such as data collected on paper forms at the point of vaccination and later manually entered into an electronic repository where they are available for reporting. In settings where resident registries are available, the use of data linkages can expedite the input process.

Some Canadian health regions chose to collect pandemic (H1N1) 2009 influenza immunization data using the same system implemented during the annual seasonal influenza vaccination campaign while in other areas, new methods were adopted. The use of a specific data collection system by a health region is typically based on numerous factors including anticipated willingness to adopt new technology as well as financial and human resources. An influenza pandemic offers a unique opportunity to study the relative efficiency of these data collection processes across numerous settings all responding to the same high profile health event. A time comparison of immunization data collection methods would provide invaluable information to healthcare decision-makers who are reviewing their future plans after this recent pandemic as well as for public health authorities involved with any mass vaccination campaign.

Time and motion studies involve an observer following the movements and tasks of an individual and recording the amount of time that is taken to complete the activity, during a typical work shift. While observer-induced bias can occur, it can be reduced by taking steps to reduce the conspicuousness of the observer[2]. Studies are best carried out in an environment that prevents the observer from altering the task completion, but allows for accurate data collection of the time spent completing each task. While potentially expensive and time-consuming, time and motion studies allow for the most accurate measurement of structured components; therefore this design is ideal for evaluating the efficiency of an immunization visit which is typically comprised of standard, defined tasks[3].

As members of the Public Health Agency of Canada/Canadian Institutes for Health Research Influenza Research Network (PCIRN)'s Vaccine Coverage Theme, our mandate is to help address identified research gaps in Canada's pandemic influenza preparedness initiative. Our research is directed at identifying and evaluating approaches to collecting pandemic vaccine delivery data at the point of care (the theme's evaluation framework is presented in Table 1). In this paper, we describe the protocol developed to meet the following objectives: i) to observe how pandemic immunization data collection methods affect a clinic's staffing requirements, equipment requirements and immunization process; ii) to measure the time spent by front-line immunization staff (physicians, nurses, administrative support) to record client data for pandemic immunization in public health clinics and healthcare institutions using electronic, paper-based or hybrid data collection systems and iii) to identify the perceptions and attitudes of frontline pandemic (H1N1) 2009 influenza immunization staff toward data collection methods employed at their sites.

**Table 1:** PCIRN Vaccine Coverage Theme - Evaluation Framework

Attribute	Description of Measures
Simplicity	Ease of operation for vaccine delivery and support staff to collect immunization data, and for decision makers and planners to obtain the information they need for monitoring purposes.

Flexibility	Capacity to accommodate modifications to reflect changing requirements and local needs.

Data Quality	Completeness (absence of missing data elements) and validity (absence of errors in the data) of the data recorded, and suitability of the data for satisfying reporting requirements. Suitability for research purposes.

Acceptability	Willingness of persons and organizations to use the immunization data collection system, as well as feasibility of adoption.

Representativeness	Accuracy in describing vaccine coverage over time and its distribution in the population by person and place (i.e., ensuring no exclusion of selected population subgroups or geographical areas).

Sensitivity	Proportion of vaccine recipients captured in data collection system (since immunizations taking place across numerous settings may lead to incomplete data capture).

Timeliness	Time required from act of immunization to generation of vaccine coverage estimates.

Stability	Reliability (ability to collect, manage, and provide data properly without failure) and availability (ability to be operational when it is needed) of the immunization data collection system.

Security	Presence of processes and mechanisms to protect the privacy and confidentiality of client information and to prevent data loss.

## Methods/Design

### Study Design

The study was composed of a three-stage evaluation comprised of passive clinic observation, a time and motion study and survey methodology to assess data collection approaches used in pandemic (H1N1) 2009 influenza vaccination sites across Canada. Site visits occurred between October and December 2009, during Canada's pandemic vaccination campaign.

### Site Inclusion Criteria

Participating clinics were public health sites or healthcare institutions, selected to include representation from each province and territory, as well as urban and rural settings. However, given that the pandemic vaccine was only to be offered in mass vaccination clinics for a finite amount of time, we anticipated that all sites that agreed to participate would be selected as study sites and did not define a specific sample size.

### Recruitment

Email invitations were sent to Medical Officers of Health of health regions and other provincial and regional health authorities across Canada as well as local public health department managers. The emails invited recipients to have their health authority or clinic participate as a study site, to propose alternate study sites under their jurisdiction that may be interested in enrolling, or to request more information regarding the study. Follow-up telephone calls were also made. Once a site was recruited, the site manager and research team decided on three specific evaluation days for the visit. One study research associate (RA) attended each study site for the three-day observation period. At the beginning of each of the three observation days, health region staff members had the opportunity to ask the RA any questions about the study. The primary inclusion criterion for participation in both the time and motion and perception survey stages was that the frontline staff member had to be involved with the data-collection related aspects of an immunization visit. Participating staff members also had to be proficient in reading and writing in either English or French (all study forms were available in both languages). Staff members who wished to participate were asked to provide written consent before they were observed and timed.

A notice was provided at all study sites, either on the registration desk or posted at the site entrance, to notify clients that the site was participating in a research study. The notice also indicated that clients were under no obligation to have an RA observe their visit and they could verbally opt out without any consequence to their level of care.

*Stage 1 (Passive Clinic Observations) *- On the initial observation day at a site, the RA spent the first 1-2 hours passively observing the clinic to become familiar with the immunization processes as well as the specific core immunization tasks that each staff member was responsible for at that site. It was expected that the following five core tasks comprised the aspects of an immunization visit where clinic staff members collected/recorded client data:

(i) Client registration/check-in: client's demographic information (name, sex, date of birth, postal code, health number etc.) was collected

(ii) Medical history collection: client's risk status as well as history of chronic conditions and previous seasonal influenza vaccine administration was collected

(iii) Medical history review: client's medical history was reviewed and additional information was recorded, as necessary

(iv) Vaccine administration record-keeping: after the vaccination, pertinent information regarding the administration (vaccine, dosage, dose number, lot number, vaccinator's name, site of vaccination etc.) was recorded

(v) Preparation of proof of vaccine administration for the client: a paper record was created indicating that the client had been vaccinated

During this clinic observation stage, the research team noted the clinic layout and data collection processes used for the core tasks and recorded any other general observations. The research team used the H1N1 Immunization Clinic Data Collection Methods Observation Guide [4] to steer the data collection (Additional File 1). The team also identified the verbal/visual cues that denoted the start and end of each task, in preparation for Stage 2, the time and motion study. Examples of potential cues are defined in Table 2, but differed depending on the clinic's layout and processes. In cases where two or more tasks were completed simultaneously, making it difficult to get accurate measurements of each task separately, total times for these tasks were measured and recorded accordingly. Days 2 and 3 began with the clinic observation stage for a shorter duration, as the majority of data collected did not vary from day to day at a location.

**Table 2:** Examples of Start/End Cues for Immunization Tasks

	Electronic Systems		Paper/Hybrid Systems	
	
	Start Cue	End Cue	Start Cue	End Cue
**Task 1**: *Client Registration*	The client hands the health card to the registration staff member for swiping	The staff member stops confirming the demographic information that is entered into the system	The staff member begins asking the clients for demographic information	The staff members puts his/her pen down following collecting data

**Task 2**: *Medical History Collection*	The staff member begins asking the client about risk status	The staff member direct the client to the vaccination area	The staff member begins asking the client about risk status	The staff member direct the client to the vaccination area

**Task 3**: *Medical History Review*	The client sits with the nurse at the vaccination area	The nurse appears to have stopped asking questions/is no longer typing	The client sits with the nurse at the vaccination area	The nurse appears to have stopped asking questions/is no longer typing

**Task 4**: *Vaccine Administration Record-Keeping*	Nurse checks off computerized form for record keeping	Nurse switches to next form	Nurse picks up pen to record details of the vaccination	Nurse puts his/her pen down

**Task 5**: *Preparation of Proof of Vaccine Administration for Client*	Nurse types in proof of vaccine administration (name, date, vaccine name)	Nurse tells client the visit is complete	Nurse picks up pen to complete the proof form	Nurse hands the form to the client

*Stage 2 (Time and Motion Study) *- The RA timed the completion of each of the five core tasks by observing consenting staff members who had a core task responsibility. For example, when each of the five tasks was completed by a separate staff member, the RA observed five staff members who complete these tasks and consented to the study. Based on the layout of the clinic, the RA tried to choose an unobtrusive location to observe the staff member close enough to the interaction to see and/or hear it take place (to record its duration) but at a great enough distance to not interfere with the process. Start and end times were based on both visual and verbal cues which were recorded. For example, the "start time" of an observation may have been the initial registration-related task for a new client (i.e. began a new data form, engaged the client in related conversation). "End time" was defined as the point when the registration person had completed all tasks associated with that specific client's registration. The RA utilized a tablet computer and recorded observation times on an electronic Data Collection Form (which included a time-stamp function that was used to time the tasks). When the use of electronic devices was not feasible at a study site, the RA timed and recorded task completion using a stopwatch and paper forms, respectively. For each of the three days, the RA observed and timed the completion of 20 consecutive client interactions for each of the five core tasks, where possible. Therefore, at the end of the three evaluation days, there were 60 timed measurements for each of the five tasks.

In order to optimize the probability that a "typical day" was being captured by the measurements, and recognizing that there may be different levels of throughput at different times of the week, the RA attempted to attend the immunization site on 3 different days of the week. The RA also tried to conduct the observations at different times on each of the three days, to account for time-based differences in client-throughput as well as staffing. In some instances, it was only possible for the RA to attend a clinic for two days rather than three (due to logistical issues or clinic schedules); therefore, on one of the two days, the RA timed 40 observations per core task, rather than 20, for a total of 60 observations per task over both days. Additionally, when clinics did not operate at a single location for more than one or two days, it was necessary for the RA to conduct the three-day evaluation at multiple sites within a single region.

*Stage 3 (Staff Perception of Data Collection Methods) *- On each day, after observation of the 20 client interactions per task, the observed staff member completed and returned the Perceptions of Pandemic (H1N1) 2009 Influenza Data Collection Methods Questionnaire. This Likert scale questionnaire was developed to elicit staff user perceptions of the data collection method employed at the study site (Additional File 2). On the anonymous questionnaire, staff members were asked to provide demographic information including any professional designation, role and the number of years they had used the method employed at their site. On Day 1 only, observed staff members were also asked to complete the Collected Data Elements Questionnaire, to indicate which data elements they routinely collected from the client during their core task (Additional File 3).

### Pilot Study

A small pilot study was conducted in a convenience sample of three seasonal influenza vaccination clinics in Ontario, immediately before the pandemic (H1N1) 2009 influenza vaccination campaign began across Canada. The purposes of the pilot study were to test the proposed methodology for the time and motion study (Stage 2), to ensure that it was logistically feasible, to provide the opportunity for any necessary modifications before the full study began and to determine inter-rater reliability in the measurements of the RAs who observed and timed the immunization staff.

The RAs attended each pilot study site for a 1-day evaluation period during which they tested out study methodology for each of the three stages, including the use of all data collection forms. To pilot Stage 2, the RAs timed the completion of each of the five core tasks by observing staff members who had a core task responsibility. For example, when each of the five tasks were completed by a separate staff member, the RAs chose five staff members who completed these tasks and independently observed each one in turn over the course of the same client interactions. For the purpose of this pilot study, each staff member being observed was timed independently by the RAs for three observations each. Following all three observations of one staff member, the RAs compared timing results. When the time duration for each observation varied by less than 5 seconds, the RAs continued on to observe and time the next core task. If the variation between two or more of the RAs' measurements was greater than five seconds, the RAs discussed their timing process in order to reach a consensus on the visual and verbal cues they used to assume "start" and "end" times. They then repeated the process for additional observations, until the durations were within five seconds of each other. At this point, the RAs observed a staff member who was completing the next core task. The threshold of five seconds was chosen as a sufficiently short duration to ensure adequate reliability, given that immunization tasks could potentially be completed fairly quickly. To assess inter-rater reliability the intraclass correlation coefficient was calculated for each task in four of the six raters. Bland and Altman plots were constructed for each task to assess agreement between the raters with the maximum and minimum times.

At completion, the research team noted any changes to their timing process and to the data collection forms that were required for the full study in order to ensure satisfactory study flow as well as minimal disruption to the immunization process.

### Data Management

All data were collected using standardized paper or electronic data forms and transferred into a shared Microsoft Excel 2007 file at the end of each site visit. This file is password-protected and can only be accessed by members of the research team. Collation and data analysis is being carried out by the research team at the Ontario Agency for Health Protection and Promotion, Ontario.

### Statistical Analysis

*Stage 1 (Clinic Observations)*: The results of the clinic observation component will be analyzed descriptively, and compared across data collection methods.

*Stage 2 (Time and Motion Study)*: Based on the above methodology, it is expected that observation time will be calculated for each separate component of the vaccination process. However, in the event that two or more tasks were completed simultaneously, making it difficult to distinguish time per task, a total vaccination time will be determined for clients, constituting a sum of those tasks. The mean observation time per task across all sites per data collection method will be calculated. Average times will be compared across data collection methods using an analysis of variance (ANOVA) test. The outcome will be assessed for parametric assumptions, and if these are not met, the Median test, the Kruskal-Wallis H test, and the Mann-Whitney *U *test will be used. Covariates to be investigated in the model include client throughput, clinic population, location, vaccination week, RA and staff member. Clinic times and percentage of data collection elements collected will be compared across type of study site by data collection method. Statistical models will be fit using SAS version 8.1 statistical software (Cary, North Carolina) per core task, as well as in total across all five tasks.

*Stage 3 (Staff Perception of Data Collection Methods)*: Univariate ANOVA will be used to compare responses based on data collection method. Should a statistically significant result be obtained (p < 0.05), Tukey's pairwise comparison will be used to determine where the differences existed. Responses will also be analyzed and summarized by type of study site. Clinic times and percentage of data collection elements collected (from full set in Additional File 3) will be compared across type of study site by data collection method.

### Ethical Considerations

This project has ethics approval from the University of Toronto Research Ethics Board. When potential sites required additional approval from their own ethics board before participating, this was sought if an expedited review was available. If REB approval could not be obtained before the end of the pandemic vaccination campaign in that region, the site was not able to participate. No identifying clinic staff or client information was collected during this study.

## Discussion

We report the protocol of a study that was developed to compare the different approaches that were used to collect pandemic (H1N1) 2009 influenza immunization data across Canada. To our knowledge, this is the first study to compare electronic, hybrid and paper-based immunization data collection systems based on the outcome measures of time and user perception.

Public health regions can vary tremendously in terms of population size, demographics, health status and other characteristics and it cannot be expected that a single immunization data collection system will be optimal across these regions. This study is based on a convenience sample of health regions which may limit its representativeness and generalizability. However, we attempted to recruit a cross-section of different health regions. At the completion of data analyses, each participating health region will receive feedback on their clinic's performance, relative to other similar health regions. A broader report, comparing manual, electronic and hybrid systems of influenza immunization data collection but not specifically identifying participating sites or provinces will be disseminated to share findings with all public health jurisdictions.

Comparing data collection approaches at immunization clinics across the country using time and motion studies is an appropriate way of evaluating the efficiency of such methods, which will provide valuable information to pandemic planners and public health authorities planning mass immunization responses. Additionally, results of this study will guide public health decision-makers at all government levels to improve planning while conducting mass immunization campaigns and when creating new data collection methods or modifying current processes for both influenza vaccination and other public health interventions.

## Abbreviations

ANOVA: Analysis of Variance; RA: Research Associate.

## Competing interests

The authors declare that they have no competing interests.

## Authors' contributions

JAP, SQ, CH, JF, ShQ and JCK participated in the design of the study with contributions from MF, CAS, JAB, DLB, AM and SD. JAP, SQ, CH, JF, ShQ and JCK carried out the study and drafted the manuscript. All authors reviewed and approved the study protocol manuscript.

## Pre-publication history

The pre-publication history for this paper can be accessed here:

http://www.biomedcentral.com/1471-2288/10/51/prepub

## Supplementary Material

Additional file 1 **H1N1 Immunization Clinic Data Collection Methods Observation Guide Guide used to direct the passive observation component of the study**. Click here for file

Additional file 2 **Perceptions of Pandemic (H1N1) 2009 Influenza Client Data Collection Methods Questionnaire Questionnaire to elicit perceptions of data collection method employed at the respondent's clinic**. Click here for file

Additional file 3 **Collected Data Elements Questionnaire Questionnaire to determine what data are collected at the respondent's clinic, as well as key functionalities of the system employed**. Click here for file
