# Supplementary material for: Pan-Canadian assessment of pandemic immunization data collection: study methodology
Source: BMC Med Res Methodol. 2010 Jun 8;10:51. doi: 10.1186/1471-2288-10-51 (PMC2896946; doi:10.1186/1471-2288-10-51)
Supplement: Additional file 1 — H1N1 Immunization Clinic Data Collection Methods Observation Guide Guide used to direct the passive observation component of the study. [file 1471-2288-10-51-S1.DOC]

**Appendix I: H1N1 Immunization Clinic Data Collection Methods Observation Guide**

- Employ a passive method to record what you see, feel, hear or smell; this does not include asking questions of the people you are observing, but may include comments or exchanges they make within your hearing.
- Observe what you can, but do not intrude at any time on client privacy or provider/client interactions.
- Review the suggested questions and record notes on aspects observed.
- Please record your field notes as soon as possible after making the observations.
- Please do not record any names in your field notes.
- Field notes are confidential and will not be read by anyone beyond the Research Team for this project, and will be reported only in aggregate form.

*In your field notes, consistently label the clients and staff that you are noting. Use the following code system: CM1 = Client Male 1, CF2 = Client Female 2, etc.; SF1 = Staff Female 1, SF2 = Staff Female 2, etc. Include a legend in your field notes for all other codes.*

Directions:

Present yourself to the clinic staff, on your arrival at the clinic and indicate that you are there as a member of the Evaluation Team to make process observations for use in the evaluation of the project.

*During your shift(s) as an* ***Observer*** *consider the following Process Evaluation Questions as you record your observations:*

Look at the work environment: what is the staff doing; specifically, what did you see?

Are there any comments made about working in the clinic; what are these comments?

Did staff members seem to know the roles they were supposed to play?

Did staff members seem to know the procedures they were to follow?

Did staff members seem to have received adequate orientation to the data collection system?

Did you note any barriers to staff/physicians performing their duties?

Record the number of different members of staff each client sees in a visit; the number on staff on site- is it adequate?

What data collection equipment is being used (e.g. are the computers old?)

Does the staff appear to be struggling with the data collection method?

Does there appear to be capacity to handle the clients?

Does there appear to be communication between the clinic stages?

Comments or other indications by staff on the convenience, comfort, etc. in the clinic?

What seems to be working well, and why? What doesn’t seem to working, and why?

**Page 2**

**H1N1 Immunization Clinic Data Collection Methods Observation Guide**

**Confidential**

Ethnographic Data Collection

**Date of visit: Time of visit: to**

**Type of Clinic: Location of clinic:**

**Observer’s name:**

**Questions for the Clinics Manager:**

1. **How many hours is the clinic operating today?**
2. **How many clients do you expect to vaccinate within this time?**
3. **If this is a hybrid system (manual then electronic batch data entry), how often does the batch data entry take place?**
4. **If data are captured manually first, how long does it take to enter one form electronically?**
