# Supplementary material for: Pan-Canadian assessment of pandemic immunization data collection: study methodology
Source: BMC Med Res Methodol. 2010 Jun 8;10:51. doi: 10.1186/1471-2288-10-51 (PMC2896946; doi:10.1186/1471-2288-10-51)
Supplement: Additional file 2 — Perceptions of Pandemic (H1N1) 2009 Influenza Client Data Collection Methods Questionnaire Questionnaire to elicit perceptions of data collection method employed at the respondent's clinic. [file 1471-2288-10-51-S2.DOC]

**Appendix II:** **Perceptions of Pandemic (H1N1) 2009 Influenza**

**Client Data Collection Methods Questionnaire**

The Public Health Agency of Canada/Canadian Institutes of Health Research Influenza Research Network (PCIRN) is conducting a questionnaire to evaluate the different systems being used across Canada to collect pandemic (H1N1) 2009 influenza client immunization data. To collect this client data, you may be using an electronic system, a purely paper-based system, or a paper system with a later transfer into an electronic system.

We would appreciate if you would complete the below survey, which will take you approximately 5 – 10 minutes. By completing this questionnaire, you will be providing your opinions regarding the system you are using. Your responses will help us understand what aspects of the method you are satisfied with and the aspects that could be improved upon to facilitate data collection.

**Please indicate your position, by checkmark below:**

 Administrative staff

 Physician

 Vaccination nurse

 Post-vaccination nurse

 Other; please specify:

**Please indicate your responsibilities, by checkmark below (more than one may apply):­­­­­­­­**

 Client registration

 Medical history collection

 Medical history review

 Vaccine administration record-keeping

 Preparation of Proof of Vaccine Administration

**Please indicate the number of years you have had the above responsibility(ies) during seasonal influenza seasons:**

**Please indicate your site type, by checkmark below:**

 Public health site

 Hospital or other healthcare institution

 Physician office

 Other

**City: Province:**

**Please indicate the type of Data Collection Method used at your site, by checkmark below:**

 Electronic system

 Paper-based system

 Hybrid (paper-based system, transferred into electronic form)

**Number of years you have used this data collection method at your site:**

**Perceptions of Pandemic (H1N1) 2009 Influenza**

**Client Data Collection Methods Questionnaire (page 2)**

As you read each statement, please consider all of the tasks that you complete at your immunization site, using your data collection method. Then, **indicate your level of agreement with the statement by circling the appropriate response**. If a statement does not apply to you, please circle N/A.

1. **It was easy to use this data collection method.**

Strongly Disagree Neither Agree Agree Strongly N/A

Disagree nor Disagree Agree

1. **I could effectively complete my tasks using this method.**

Strongly Disagree Neither Agree Agree Strongly N/A

Disagree nor Disagree Agree

1. **I was able to complete my tasks quickly using this method.**

Strongly Disagree Neither Agree Agree Strongly N/A

Disagree nor Disagree Agree

1. **I felt comfortable using this method.**

Strongly Disagree Neither Agree Agree Strongly N/A

Disagree nor Disagree Agree

1. **It was easy to learn to use this method.**

Strongly Disagree Neither Agree Agree Strongly N/A

Disagree nor Disagree Agree

1. **Whenever I make a mistake using this method, I can recover easily and quickly.**

Strongly Disagree Neither Agree Agree Strongly N/A

Disagree nor Disagree Agree

1. **Overall, I am satisfied with this method.**

Strongly Disagree Neither Agree Agree Strongly N/A

Disagree nor Disagree Agree

**Please return your completed questionnaire to the Research Associate. Thank you!**
