# Supplementary material for: Pan-Canadian assessment of pandemic immunization data collection: study methodology
Source: BMC Med Res Methodol. 2010 Jun 8;10:51. doi: 10.1186/1471-2288-10-51 (PMC2896946; doi:10.1186/1471-2288-10-51)
Supplement: Additional file 3 — Collected Data Elements Questionnaire Questionnaire to determine what data are collected at the respondent's clinic, as well as key functionalities of the system employed. [file 1471-2288-10-51-S3.DOC]

**Appendix III: Collected Data Elements Questionnaire**

**How do you collect client immunization data at the point of care: **

 Electronic system  On paper  On paper, later transferred to electronic system

**Is a login and password required to access the system?**

 Yes  No

Please indicate with an “X” how you capture the following elements:

| **Data Element** | **Collected** | **Not Collected** |
| --- | --- | --- |
| Name |  |  |
| Unique identifier (e.g. health card number) |  |  |
| Sex |  |  |
| Date of birth |  |  |
| Ethnicity |  |  |
| Residential address (including postal code) |  |  |
| Phone number and/or email address |  |  |
| Pregnancy |  |  |
| Chronic medical condition(s) |  |  |
| Health care worker status |  |  |
| Aboriginal status |  |  |
| Contraindications to influenza vaccines |  |  |
| Prior receipt of 2008/09 and/or 2009/10 seasonal vaccines |  |  |
| Vaccinator name |  |  |
| Vaccinator affiliation |  |  |
| Vaccine name/ type |  |  |
| Vaccine dose number (for the pediatric population) |  |  |
| Vaccine dosage |  |  |
| Vaccine lot number |  |  |
| Vaccine expiry date |  |  |
| Date of immunization |  |  |
| Vaccination site (e.g. left deltoid) |  |  |
| Adverse events following immunization |  |  |

**If you have an electronic system, please indicate with a checkmark if your system has any of the following:**  Swipe card

 Pre-populated client data

 Multiple client entry

 Ability to access data in real-time

 Ability to record more than one vaccine per visit

 Rapid retrieval of client data

**Does your clinic identify high risk/ target groups at registration?**

 Yes  No

**Please return your completed questionnaire to the Research Associate. Thank you!**
